# Construction of 2-2 Type Cement-Based Piezoelectric Composites’ Mechanic–Electric Relationship Based on Strain Rate Dependence

**DOI:** 10.3390/ma16072702

**Published:** 2023-03-28

**Authors:** Haiwei Dong, Zhe Li, Ziye Zhu, Yi Li, Wenjie Cheng, Jiangying Chen

**Affiliations:** 1 Key Laboratory of Impact and Safety Engineering, Ningbo University, Ministry of Education, Ningbo 315211, China; 1801081010@nbu.edu.cn (H.D.); 2101090010@nbu.edu.cn (Y.L.); 2 Zhejiang Provincial Engineering Research Center for the Safety of Pressure Vessel and Pipeline, Ningbo University, Ningbo 315211, China; 3 Faculty of Mechanical Engineering and Mechanics, Ningbo University, Ningbo 315211, China; 4 BYD Automotive Engineering Research Institute, Shenzhen 518118, China

**Keywords:** cement-based, piezoelectric ceramic, strain rate, stress–strain relationship, mechanic–electric response

## Abstract

It has been found that the mechanic–electric response of cement-based piezoelectric composites under impact loading is nonlinear. Herein, we prepared a 2-2 cement-based piezoelectric composite material using cutting, pouring, and re-cutting. Then, we obtained the stress–strain and stress–electric displacement curves for this piezoelectric composite under impact loading using a modified split Hopkinson pressure bar (SHPB) experimental apparatus and an additional electrical output measurement system. Based on the micromechanics of the composite materials, we assumed that damage occurred only in the cement paste. The mechanical response relationship of the piezoelectric composite was calculated as the product of the viscoelastic constitutive relationship of the cement paste and a constant, where the constant was determined based on the reinforcement properties of the mechanical response of the piezoelectric composite. Using a modified nonlinear viscoelastic Zhu–Wang–Tang (ZWT) model, we characterized the stress–strain curves of the piezoelectric composite with different strain rates. The dynamic sensitivity and stress threshold of the linear response of the samples were calibrated and fitted. Thus, a mechanic–electric response equation was established for the 2-2 type cement-based piezoelectric composite considering the strain rate effects.

## 1. Introduction

Concrete is one of the most widely used materials in civil engineering construction. In addition to conventional static loads, concrete structures may also be subjected to dynamic loads such as earthquakes, typhoons, and explosions [1,2]. In many civil engineering structures, smart materials are used for online long-term non-destructive health detection and for implementing early precautionary measures. Self-perception of material properties is crucial for developing health monitoring systems with digital features. Research on the composition of self-sensing concrete has considered factors such as preparation, testing and characterization, performance and regulation, mechanism and modeling, engineering application, and so on [3]. For health detection in civil engineering structures, typically, a large number of sensing elements are attached or embedded in sensing distribution arrays. These impart sensing characteristics to the important components or integral structures of the structure. Thus, achieving real-time monitoring and safe early warning of the internal stress conditions of building structures using contemporary intelligent detection systems is a crucial research topic, and this is achieved using online health monitoring and prediction [4,5,6].

The monitoring and damage identification of structures are mainly carried out using active detection and passive monitoring technologies. Active detection technologies transmit excitation signals through actuators installed in the structures; these signals are received by sensors installed in the structures. The health state of a structure is evaluated according to the relevant physical parameters of structural vibration in combination with the relevant identification methods. Dynamic stress measurement using smart sensors is a passive monitoring technology, and it is of two types. One uses piezoelectric smart materials that are attached to surfaces or embedded as actuators and sensors; it carries out real-time monitoring by transforming fluctuation signals in the materials. Another type uses intelligent sensors for monitoring the mechanical impedance of materials for structural health monitoring and damage identification [7,8]. To ensure accurate and effective signal monitoring, both active and passive structural monitoring technologies have high performance requirements for intelligent sensors. Traditional smart materials include piezoelectric ceramics, memory alloys, electric-magnet strictive materials, electric-magnet fluidic variant materials, and so on. However, they have some obvious drawbacks, such as acoustic impedance matching, thermal expansion differences, and interface adhesion between the smart materials and concrete surfaces. There are also deformation coordination problems during measurement, such as distorted signals that affect the measurements recorded by sensors, even leading to monitoring errors [9,10,11]. If these smart materials are employed directly, distortion signals produced by the inconsistent deformation easily affect the sensing accuracy during measurement.

According to the concept of “connectivity” between the components of composite materials, as proposed by Newnham et al. [12], cement-based two-phase piezoelectric composites are prepared using cement or mixtures thereof as the matrix, with the piezoelectric ceramic as the functional body. This can address the issue of deformation coordination between piezoelectric materials and concrete structures. Under compression loads, cement-based piezoelectric composites exhibit excellent mechanic–electric response characteristics. Sensors that are composed of a cement-based piezoelectric composite material embedded in a concrete structure have high sensing accuracy, excellent output signals, and fast response speeds, and they enable the sensing of strain and stress, as well as damage such as micro-holes and micro-cracks.

Since cement-based piezoelectric ceramic composites were first prepared in 2001, several studies have investigated their dielectric, piezoelectric, and impedance properties for different fabrication methods and processes. Li, Zhang et al. [13,14] first used ordinary Portland cement and a cement polymer (a mixture of cement, epoxy resin, and curing agent) as the matrix material, and PZT as the functional phase, to create a cement-based piezoelectric composite 2-2 type material. The sensing and driving properties of the material exhibited an obvious frequency dependence. Cheng, Huang, Liu et al. [15] used sulfoaluminate cement as the matrix and niobium–magnesium–zirconate lead P(MN)ZT as the piezoelectric functional body to prepare 1-3, 0-3, and 2-2 type cement-based piezoelectric composites via cutting and filling. They examined the influence of important factors, such as the width to thickness ratio of piezoelectric ceramics, volume fraction of piezoelectric ceramics, different matrix and functional bodies, mixing amount, curing temperature, and humidity, on the piezoelectric and dielectric properties of the three types of piezoelectric composites. Pan, Chaipanich et al. [16,17,18] examined the influence of the content of cement-based piezoelectric materials, such as graphene and carbon nanotubes/clay, on the piezoelectric, thermal, and mechanical properties and hydration. Cement-based piezoelectric sensors and PZT sensors have been used to monitor the stress–strain relationships of concrete based on electromechanical impedance.

In recent years, there has been extensive research on the dynamic properties of piezoelectric ceramics and cement-based piezoelectric composites. Lei et al. [19] determined that the mechanic–electrical response characteristics of PZT piezoelectric ceramics are considerably influenced by impact loading. An obvious rate correlation and a viscous effect of piezoelectric ceramics was observed. For piezoelectric ceramics, Chure et al. [20] observed that the output voltage and electrical energy generated increased with an increase in mechanical energy/impact loadings. Wang, Tang et al. [21] studied the stress–strain states under different strain rates using uniaxial compression and SHPB tests, with the nonlinear viscoelastic constitutive equation and piezoelectric equation; they established the PZT-5H force–electric response model considering the strain rate effect. Han et al. [22] adopted the displacement method to conduct dynamic analyses of cement-based piezoelectric composites and demonstrated that the piezoelectric elasticity theory is applicable to cement-based piezoelectric composites under different loads. Zhang et al. [23] proposed a dynamic characteristic analysis model of 2-2 type multilayer cement-based piezoelectric composites under impact loading. Based on the theory of piezoelectric elasticity, the theoretical solution to a specific problem was obtained according to the Duhamel integral and variable separation method. Zhang, Xu et al. [24,25] used the SHPB device to carry out impact compression tests for type 1-3 cement-based piezoelectric composites. There was a significant strain rate effect on the material and a critical value point in the stress–electrical displacement response curve. Existing experimental studies [19,20,21,22,23,24,25] show that the stress–strain–electrical responses of cement-based piezoelectric composites under impact loads is nonlinear, but there is a lack of reliable models to describe its characteristics, which affects the accurate calculation of the material’s mechanic–electric effect and damage evolution behavior, as well as the material design and application product design of cement-based piezoelectric composites.

In this study, a 2-2 type cement-based two-phase piezoelectric composite was prepared using cement paste as the matrix and PZT-5H piezoelectric material as the functional body. The dynamic response characteristics of the 2-2 type cement-based piezoelectric composites were studied using a modified SHPB test device, so as to determine whether the sample could maintain effective mechanical–electrical response characteristics under impact loads and the applicable range of the corresponding sensor. Based on the study of the strain rate effect and damage-softening effect, the dynamic damage viscoelasticity equation was developed. This work can provide theoretical guidance for the design and application of cement-based piezoelectric composites.

## 2. Methods

### 2.1. Sample Preparation

**Figure 1:**
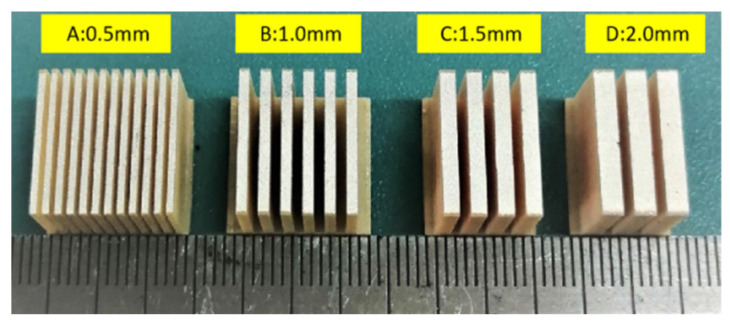
Piezoelectric ceramic sheet sizes.

In this study, a 2-2 type cement-based piezoelectric composite material was prepared by cutting, pouring, and re-cutting. The PZT-5H piezoelectric ceramic was used as the functional phase of the composite material, and ordinary Portland cement P.II.42.5 was used as the matrix phase. Adhesion between the functional phase and the matrix phase was improved using epoxy resin. First, an SYJ-400 CNC cutting machine was used to cut the PZT-5H piezoelectric ceramic. When the volume fraction of piezoelectric ceramics accounted for 31.14% of the composite materials, four piezoelectric ceramic samples A, B, C, and D were designed. The thickness and number of A–D were 0.5 mm × 12, 1 mm × 6, 1.5 mm × 4, and 2 mm × 3, respectively (Figure 1).

Cement pastes with a water cement ratio of 0.32 were poured into the piezoelectric ceramic mold and oscillated for 3 min after pouring. The poured samples were placed in standard maintenance equipment at room temperature of 20 ± 0.5 °C and humidity > 95% for 28 days. After the maintenance was complete and during the second cutting, samples with dimensions of 17 × 17 × 8 mm^3^ (length × width × height) were prepared.

### 2.2. Test Method

In 1914, Hopkinson [26] innovatively designed two bars to measure the waveforms of impact loads with time variation. Kolsky et al. [27] improved the experimental device by dividing the input bar into two parts and measuring the stress–strain relationship of materials under impact loading.

**Figure 2:**
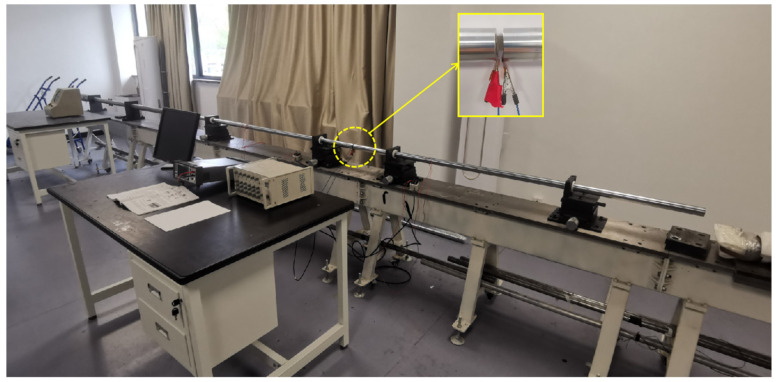
SHPB experimental setup.

The SHPB device (Figure 2) is widely applied to study the dynamic mechanical behavior of materials in the strain rate range of 10–10^3^ S^−1^ [28]. In this study, the lengths of the bullet, incident bar, transmission bar, and absorption bar were 400, 2000, 2000, and 600 mm, respectively, and the bar diameter was 37 mm. The material was high-strength spring steel with an elastic modulus of 210 GPa. To record the stress wave waveform, two sets of strain gauges were glued at the positions of the incident and transmission bars. A short sample was clamped between the incident bar and transmission bar during SHPB testing. Under a certain strike pressure, the bullet hit the incidence bar at a certain initial speed, resulting in an incident pulse load. The magnitude of the pulse load was controlled by the magnitude of the strike air pressure, and its loading duration was controlled by the length of the bullet. When the incident pulse load was transmitted to the sample, the sample deformed rapidly under the incident pulse load. The reflected pulse propagated to the incident bar, and the transmission pulse propagated to the transmission bar. A strain gauge on the incident bar was used to measure the incident and reflected wave signals, and a strain gauge on the transmission bar was used to measure the transmitted wave signal. During the experiment, the bullet, incident bar, transmitting bar, and absorber bar were all within the elastic range. The SDY2107A ultra-dynamic strainometer and a computer with programs written by ourselves were used as the acquisition, display, and storage devices for the test data.

SHPB test techniques are based on two basic assumptions [20]: (1) a one-dimensional stress wave in the bar, and (2) the uniform distribution of stress in the sample along its length. The strain rate ${\dot{\varepsilon }}_{s}\left(t\right)$, strain $\varepsilon_{s}\left(t\right)$, stress $\sigma_{s}\left(t\right)$, and average strain rate $\dot{\bar{\varepsilon }}$ of the sample were obtained using Equations (1)–(4). An available waveform pulse shaper (0.2-mm-thickness brass sheet) was added to the front of the incident bar. A trial experiment showed that it effectively decreased the waveform oscillation caused by the lateral inertia generated by the impact, and it also filtered the high-frequency components of the incident and reflected waves. For brittle materials, the rise time of the incident wave can be increased; stress uniformity was achieved inside the sample before the sample was destroyed.

(1) $$\sigma_{s}\left(t\right)=\frac{EA}{A_{0}}\left[\varepsilon_{I}\left(t\right)+\varepsilon_{R}\left(t\right)\right]$$

(2) $$\varepsilon_{s}\left(t\right)=\frac{2c_{0}}{L_{0}}\int_{0}^{t}\left[\varepsilon_{I}\left(t\right)-\varepsilon_{T}\left(t\right)\right]dt$$

(3) $${\dot{\varepsilon }}_{s}\left(t\right)=\frac{2c_{0}}{L_{0}}\left[\varepsilon_{I}\left(t\right)-\varepsilon_{T}\left(t\right)\right]$$

(4) $$\dot{\bar{\varepsilon }}=\frac{\int_{0}^{\varepsilon }\dot{\varepsilon }\left(\varepsilon \right)d\varepsilon }{\varepsilon }$$

where *A, E,* and *c*_0_ are the cross-sectional area, elastic modulus, and elastic wave velocity in the bar, respectively, and *A*_0_ and *L*_0_ are the cross-sectional area and length of the sample, respectively. The subscripts *I*, *R*, and *T* indicate the incident, reflection, and projection of stress waves, respectively.

**Figure 3:**
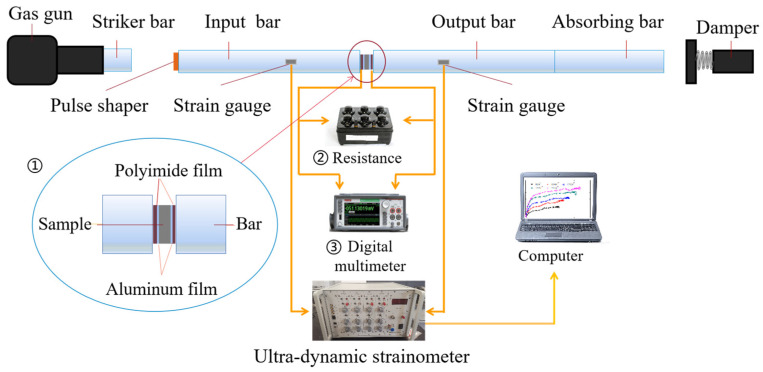
Schematic diagram of the SHPB device and the electric signal output system of the piezoelectric composite.

As illustrated in Figure 3, ➀, ➁, and ➂ constitute an additional electrical signal output system of piezoelectric materials. In the impact compression test, a high voltage of several kilo-volts was instantly generated due to the piezoelectric effect. A 100 Ω resistor was connected in parallel with the sample. The current mode circuit was used to protect the measurement circuit. The voltage *V(t)* across the resistor in the circuit was collected in real time using a Tektronix Keithley desktop digital multimeter, DMM6500. The charge discharge at both ends of the sample was determined by the current integrated in the circuit, as expressed in Equation (5) [24].

(5) $$Q\left(t\right)=\int_{0}^{t}I\left(t\right)dt=\int_{0}^{t}\frac{V\left(t\right)}{R}dt$$

The dynamic sensitivity *K* of the cement-based piezoelectric sensor was obtained using Equation (6).

(6) $$K=\frac{Q\left(t\right)}{A_{0}}\frac{1}{\sigma \left(t\right)}=\frac{C\left(t\right)}{\sigma \left(t\right)}$$

where $C\left(t\right)$ represents the electrical displacement of piezoelectric sensors.

## 3. Results and Discussion

### 3.1. Damage Evolution

**Figure 4:**
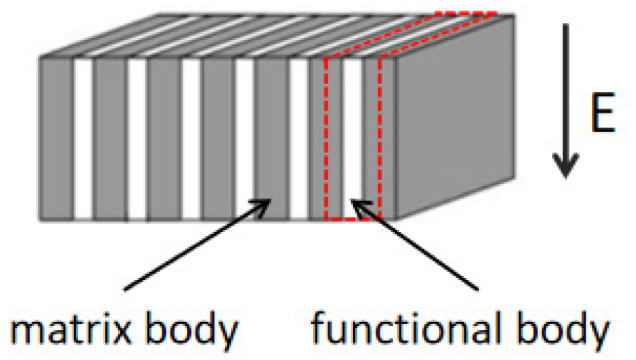
Representative volume unit.

From the perspective of micromechanics analysis [29,30,31], a representative volumetric unit in the red frame in the cement-based piezoelectric composites was taken, as illustrated in Figure 4. Based on the theory of homogenization, a constitutive equation of an ideal 2-2 type cement-based piezoelectric composite was derived. The strength of the as-developed piezoelectric ceramic was much higher than the strength of the cement matrix. Therefore, the analysis was simplified. We assumed that the damage only occurred inside the cement slurry, and its general damage evolution equation assumption was given. The strain–stress relationship of the cement-based piezoelectric composite was obtained from the product of the constitutive of the cement matrix and the constant *G*, which was determined according to the enhancement properties of the piezoelectric ceramic [32].

The dynamic destruction of the material occurred with time, and it caused different forms of micro-damage, such as micro-holes and micro-cracks, along with nucleation–growth–connectivity at finite rates [33,34]. Under shock loading, the initial micro-damage inside the cement paste nucleated and expanded, finally crushing the sample.

Damage factor *D* is a function of the strain and strain rate. When *D* = 0, there was no damage in the material. When *D* = 1, the material was completely crushed and had no bearing capacity. The strain was constant, and *D* was expressed as a power function (Equation (7)).

(7) $$D=D_{0}{\left(\varepsilon ,\dot{\varepsilon }/{\dot{\varepsilon }}_{0}\right)}^{a}\varepsilon^{b}$$

The reference strain rate was the quasi-static strain rate, ${\dot{\varepsilon }}_{0}=10^{-4}s^{-1}$. Equation (7) is expressed in a non-dimensional form. The undetermined parameter *D*_0_ is the initial damage evolution factor, which represents the micro-damage in the initial stage of the sample. The undetermined parameters *a* and *b* represent the damage influence factor, which was obtained by trial curve fitting.

Some studies [35,36] have indicated that electrical damage mainly occurs inside piezoelectric ceramics. This may be attributed to two possible causes. Firstly, under impact loading, the micro-cracks generated inside the piezoelectric ceramic form an electrical insulation boundary, which does not allow the electric charge to effectively pass, resulting in a voltage loss. Secondly, due to the generation of micro-cracks in the piezoelectric ceramic, the stress direction after damage does not follow the polarization direction. Overall, when a smaller external macroscopic charge was generated, there was a nonlinear mechanic–electric response.

### 3.2. Enhancement Effect of Piezoelectric Ceramics

Lei et al. [19] proposed that during impact loading, the strain of PZT-5H piezoelectric ceramics exhibits viscous properties, which may be related to the characteristics of domain movement. Moreover, the mechanical properties showed an obvious rate correlation. We assumed that the interface between the cement paste and the PZT-5H piezoelectric ceramic was completely bonded and that the two materials had different internal stress characteristics under impact loads. According to the parallel equivalence hypothesis, individual components in a composite material have equal strain.

The elastic modulus $E_{T}$ of the cement-based piezoelectric composite was obtained using Equation (8).

(8) $$E_{T}=\left(1-f\right)E_{C}+\gamma fE_{P}$$

where *γ* is the influence factor of the modulus, *f* is the volume fraction of piezoelectric ceramics, and *E_P_* and *E_C_* are the elastic moduli of the piezoelectric ceramics and cement pastes, respectively.

The strengthening coefficient G of piezoelectric ceramics in the cement-based piezoelectric composites was calculated using Equation (9).

(9) $$G=\left(1-f\right)+\gamma f(E_{P}/E_{C})$$

### 3.3. Dynamic Damage Physical Equation and Verification Analysis

According to the principle of Lemaitre strain equivalence in damage mechanics [34], the strain of damaged materials under the action of the equivalence force is equivalent to the strain that occurs when the same material is not damaged. According to this principle, the stress of a damaged material can be derived from the stress of an undamaged material. The damage coupling stress–strain relationship of cement-based piezoelectric composites was expressed using Equation (10).

(10) $$\sigma_{c}^{d}=\left(1-D\right)\sigma_{c}^{i}\left(\varepsilon ,\dot{\varepsilon }\right)$$

where $\sigma_{c}^{d}$ indicates the internal stress of the damaged material, and $\sigma_{c}^{i}$ indicates the internal stress of the undamaged material.

**Figure 5:**
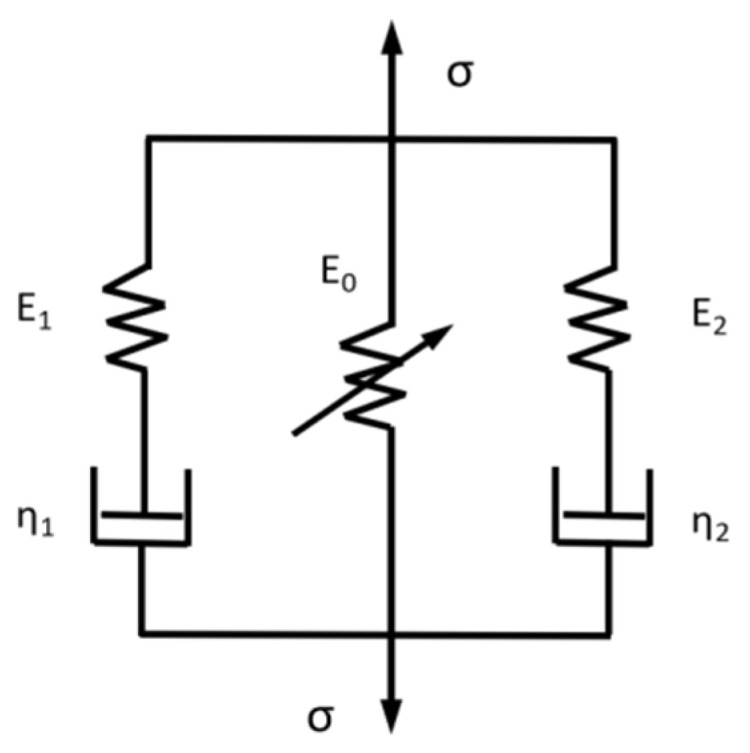
ZWT model.

For the nonlinear deformation based on the rate correlation of the internal damage evolution of the material, it was studied in the literature “A damage-modified nonlinear visco-elastic constitutive relation and failure criterion of PMMA at high strain-rates” [37]. According to the dynamic constitutive relationship of concrete materials under impact loads, the ZWT equation that calculated the damage evolution of materials was expanded for use in the cement-based piezoelectric composites (with cement pastes as the matrix). The mechanical model of the ZWT equation comprised two Maxwell models and a nonlinear spring, in parallel, as illustrated in Figure 5. In the ZWT equation, the nonlinear viscoelastic response comprised two aspects, namely a nonlinear viscoelasticity response correlated to a low strain rate and nonlinear viscoelasticity responses correlated to a high strain rate.

Combined with the theories of damage evolution and viscoelasticity, the viscoelastic mechanistic response equation of cement-based piezoelectric composites was established. The stress–strain relationship in the strain rate range of 10^−4^–10^3^ S^−1^ was described using Equation (11).

(11) $$\sigma =\left(1-D\right)G\left\{E_{0}\varepsilon +\alpha \varepsilon^{2}+\beta \varepsilon^{3}+E_{1}\int_{0}^{t}\dot{\varepsilon }\exp\left(\frac{t-\tau }{\theta_{1}}\right)dt+E_{2}\int_{0}^{t}\dot{\varepsilon }\exp\left(\frac{t-\tau }{\theta_{2}}\right)dt\right\}$$

In the above equation, the material parameters include *G*, *γ*, *f*, and *E_P_* and *E*_C_, which are the reinforcement coefficient, influence factor of the modulus, volume fraction of the piezoelectric ceramic, and elastic modulus of the piezoelectric ceramic and the pure cement pastes, respectively. *D* is given as $D=D_{0}{\left(\dot{\varepsilon }/{\dot{\varepsilon }}_{0\;}\right)}^{a}\varepsilon^{b}$, where *D*_0_ is the initial damage evolution factor; the reference strain rate ${\dot{\varepsilon }}_{0}$ is 10^−4^ S^−1^; the undetermined parameters *a* and *b* indicate the damage influence factor; *E*_0_, *α*, and *β* are the elastic coefficients of the nonlinear spring; *E*_1_ and *θ*_1_ are the modulus and relaxation time determined by the viscous response at a low strain rate, respectively; and *E_2_* and *θ*_2_ are the modulus and relaxation time determined by the viscous response at a high strain rate, respectively.

At a high strain rate, $E_{1}\int_{0}^{t}\dot{\varepsilon }exp\left(-\frac{t-\tau }{\theta_{1}}\right)dt$ was small and negligible. Therefore, only the terms at a high strain rate in Equation (11) were considered. Thus, it was simplified to Equation (12).

(12) $$\sigma =\left(1-D\right)G\left\{E_{0}\varepsilon +\alpha \varepsilon^{2}+\beta \varepsilon^{3}+E_{2}\int_{0}^{t}\dot{\varepsilon }\exp\left(\frac{t-\tau }{\theta_{2}}\right)dt\right\}$$

The law of the mechanic–electric response of the cement-based piezoelectric composite material was expressed using Equation (13), where $D_{e}$ indicates the electrical displacement, *k*_1_-*k*_4_ express the different fitting parameters, *k*_2_ represents linear sensitivity, and *M* indicates the threshold value of the nonlinear curve.

(13) $$D_{e}=\left\{\begin{array}{c} k_{4}\sigma^{3}+k_{3}\sigma^{2}+k_{2}\sigma +k_{1}\;\left(M\leq \sigma \right)\\ k_{2}\sigma +k_{1}\;(0<\sigma <M) \end{array}\right.$$

Under impact loading, the constitutive relationship of the 2-2 type cement-based piezoelectric composite material was calculated using Equations (12) and (13).

### 3.4. Verification of the Dynamic Stress–Strain Relationship

**Figure 6:**
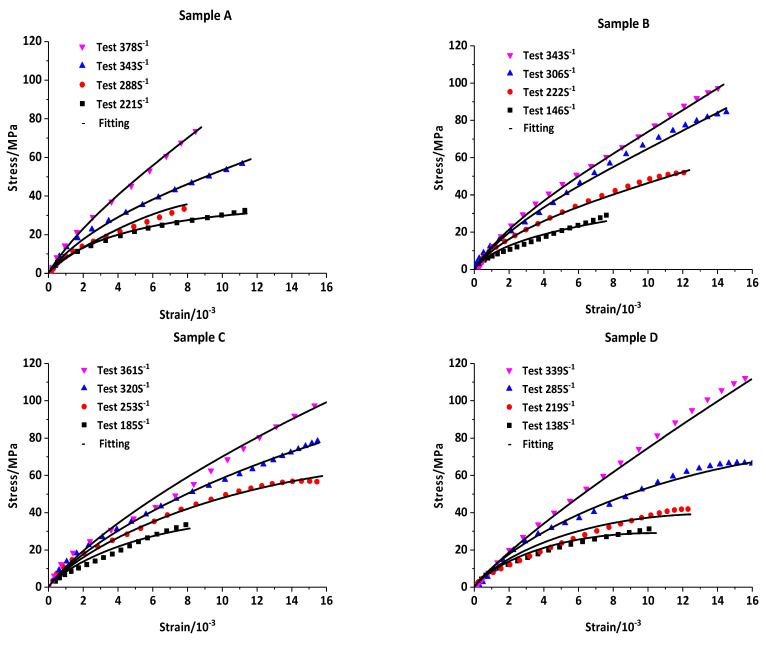
Stress–strain curves and fitting curves of samples A, B, C, and D at different strain rates.

In dynamic impact tests, by controlling the strike air pressure of the bullet, the stress–strain relationship of a 2-2 type cement-based piezoelectric composite sample was obtained under different strain rates. The trial results were analyzed and fitted using Equation (12), and the stress–strain fitting curve of the composite sample was obtained. The fitting correlation coefficient was >97.5%, and the specific results are illustrated in Figure 6.

The material fitting parameters were fitted by the trial results, as shown in Table 1. According to the fitting results of the measured curve, the influence factor of modulus *γ* = 0.2594. The fitting parameters were substituted in Equation (9), and G= 0.9780 (<1). Therefore, according to these results, in the range of approximately 150–450 S^−1^, adding the piezoelectric ceramic to the cement matrix did not significantly strengthen the matrix; however, a certain weakening effect was observed.

**Figure 7:**
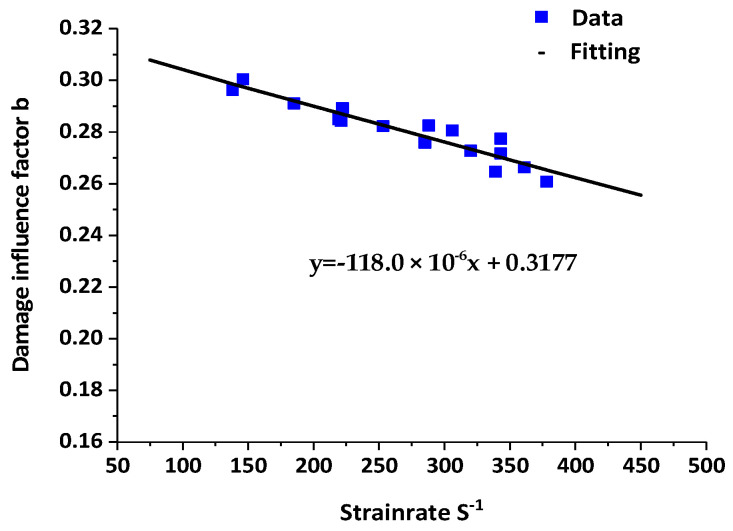
Fitting results of damage influence factor *b* and strain rate $\dot{\;\varepsilon }$.

Parameters *a* and *b* were the damage influence factors in Equation (7), respectively. The damage influence factor *a* was a fixed value of 0.0916. According to the fitting results of parameters by the experimental data, the relationship between damage influence factor *b* and different strain rates$\dot{\;\varepsilon }$ was established (Figure 7). *b* decreased with an increase in strain rate$\dot{\;\varepsilon }$, indicating the weakening influence of damage on the stress–strain relationship.

### 3.5. Mechanical and Electrical Response Characteristics

Under impact loading, electrical signals are generated by piezoelectric ceramics of cement-based piezoelectric composites. Domain switching is typically considered as the inherent mechanism of nonlinear behavior in piezoelectric ceramics. Domain motion was influenced by the loading rate, and the mechanical properties of PZT-5H piezoelectric ceramics showed an obvious rate correlation with impact loading [38,39]. The fitting curves of the mechanic–electric relationship of the 2-2 type cement-based piezoelectric composites were calculated using Equation (13). The fitting parameters of the curves are shown in Table 2. The fitting correlation coefficient was >98%.

**Figure 8:**
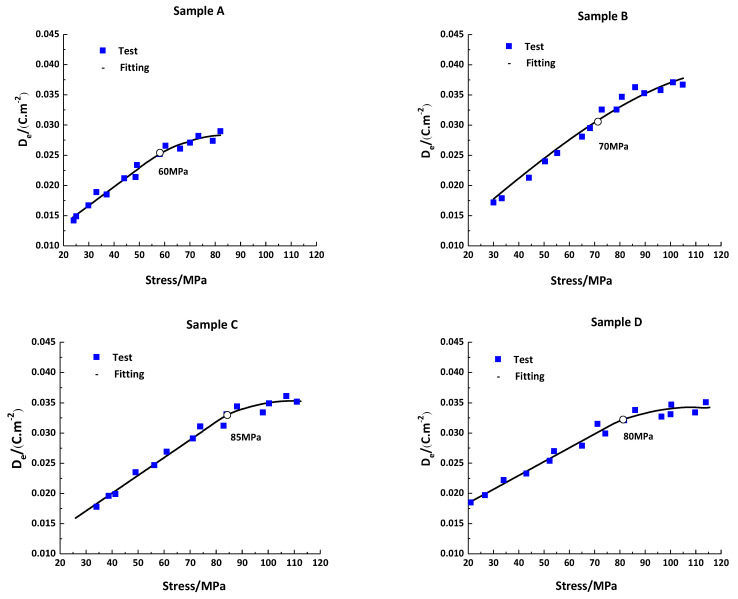
Dynamic sensitivity calibration curves of samples A, B, C, and D.

There was dynamic sensitivity and a threshold value in the mechanic–electric response relationship of the composite sample under impact compression loading (Figure 8). According to the curve fitting, for samples A, B, C, and D, the dynamic sensitivity was 311, 343, 294, and 229 pC/N, respectively. Meanwhile, the threshold values were 60, 70, 85, and 80 MPa, respectively. The linear sensitivity of sample B was 343 pC/N, and the linear threshold value of sample C was 85 MPa, which was larger than the other sample. When the loading was less than the threshold value, there was a linear relationship between the output displacement peak and the loading stress. When exceeding the threshold value, there was obvious nonlinearity.

## 4. Conclusions

In the study, a 2-2 type cement-based piezoelectric composite material was fabricated using P.II 42.5 ordinary Portland cement as the matrix and a PZT-5H piezoelectric ceramic as the functional body. The mechanic–electric response properties of the as-developed composite were tested using the SHPB device. The following conclusions were obtained.

(1) The compressive strength of the sample increased with an increase in the loading strain rate, and the material had an obvious strain rate effect.
(2) It was assumed that the damage occurred only inside the cement paste. The modified nonlinear viscoelastic ZWT equation and the nonlinear stress–electric displacement equation of the 2-2 type cement-based piezoelectric composite were established.
(3) The stress–strain curves of the 2-2 type cement-based piezoelectric composites under different strain rates were obtained using the SHPB device. The experimental results were fitted using the modified ZWT equation, and the fitting parameters of the equation were determined. With the strengthening coefficient *G* considering the piezoelectric ceramic term, the fitting result showed that the *G* value was <1 (=0.9780). The results show that piezoelectric ceramics have a certain weakening effect on the stress–strain behavior of a cement matrix when the strain rate is in the range of 150–450 S^−1^.
(4) The stress–electric displacement curve was measured using an additional electrical signal acquisition device, and the dynamic sensitivity and linear threshold of the sample were obtained by data fitting. We compared four samples A–D; the linear sensitivity of sample B was 343 pC/N, and the linear threshold value of sample C was 85 MPa, which were the largest. The curve after the linear threshold was a nonlinear response curve of a cubic polynomial. Therefore, the nonlinear relationship of the strain–stress–electric displacement of 2-2 type cement-based piezoelectric composites was established.

## Figures and Tables

**Table 1:** Fitting results of parameters using experimental data.

1	2	3	4	5
***D**_**0**_*	***a***	***γ***	***f***	***E**_**P/**_* **GPa**
0.02	0.0916	0.2594	31.14%	60.90
**6**	**7**	**8**	**9**	**10**
***Ec*** **/GPa**	***α*** **/GPa**	***β/*** **GPa**	***E**_**2/**_* **GPa**	***θ**_**2/**_* **μs**
17.00	316.50	−937.13	22.50	15.03

**Table 2:** Dynamic fitting parameters of the curve.

No.	Sample	Linearity Range /MPa	Linearity Sensitivity/(pC/N)	Dynamic Sensitivity Parameters			
				K_1_	K_2_	K_3_	K_4_
1	A	0–60	311	0.00734	0.000311	1.50 × 10^−6^	−2.65 × 10^−8^
2	B	0–70	343	0.00731	0.000343	4.79 × 10^−7^	−9.33 × 10^−9^
3	C	0–85	294	0.00829	0.000294	1.43 × 10^−6^	−1.70 × 10^−8^
4	D	0–80	229	0.0139	0.000229	9.25 × 10^−7^	−1.20 × 10^−8^

## Data Availability

The data used to support the findings of this study are available from the corresponding author upon request.
